# Extracts and Gleanings

**Published:** 1871-08

**Authors:** 


### PART II.

### Abridged Extracts and Gleanings from our Exchanges

*MULTUM IN PARVO.*

THE BROMIDE OF QUININE, MORPHINE AND STRYCHNINE are mentioned in the last number of the Practitioner, in connection with some remarks by Dr. B. W. Richardson on their therapeutic effects. These organic bromides, as he terms them, are made by the action of hydrobromic acid upon the alkaloids of these substances, or of the bromide of potassium upon their salts. All of them are soluble, and he has found it most convenient to employ them in the form of syrups made in the proportion of one grain of the quinia salt, one-fourth of a grain of the morphia salt, and one thirty-second of a grain of the strychnia salt to the drachm of syrup. They may also be combined in couples or altogether in the same proportions, one drachm of the syrup being the dose.

Speaking generally of these salts, he says: The bromide throughout, in so far as its action is indicated, is eliminative and sedative. He is satisfied that the bromide of quinine can be administered freely, where quinia itself or other of its salts cannot be tolerated. He thinks it possible to give much larger doses without producing cinchonism, than with any other of the compounds of this substance, and is of the belief that its use in the early stages of eruptive diseases would do very much to allay the severe nervous symptoms which usher them in. He has satisfied himself by experiment, that the bromide reduces, or rather subdues and prolongs, the action of strychnia on muscular motion; and lastly, a smaller dose of this salt of morphia is, in his opinion, required, than is effected in the case of the other morphia salts, and its repeated administration is not followed so certainly or quickly by those after-effects which sometimes preclude their use. This combination has had the most happy effect when administered for the purpose of calming neuralgic pain. The bromide of strychnia has been of most marked service in cases of what we sometimes call "nervous dyspepsia." He remarks that none of these remedies are admissible, however, in cases where there is much dryness and irritibility of the mucous membrane of the pharynx and larynx, on account of the long-continued cough and spasm which they induce.

CUNDURANGO, the so-called South American specific for cancer, is still a popular sensation. It is to be hoped that all the papers say of its wonderful virtues, and all that Dr. Bliss, of Washington claims for it, are true. There is, however, a little too much of the newspaper element in its notoriety to satisfy the minds of scientific men. Dr. Bliss, its principle advocate, has already become a martyr to the opinions of his skeptical professional brethren in Washington and elsewhere, but is determined to vindicate himself, and to prove the value of the remedy—at least so the daily papers inform us. In reply to an application for some of the specific, we learn also from a daily paper that he says: "I receive but a small quantity at a time, and it being my purpose to treat a few cases here, where they can be under my own observation, it will be impossible for me to send any of it away. The remedy, as well as myself, having been attacked, I desire to demonstrate to the public what it will do, which necessitates the above course."

Further on in the same newspaper paragraph he informs all ine terested that he expects to receive by the 15th of August, at the furthest, a sufficient amount of the drug "to supply the profession or the public, as they may desire." Again, in the same paragraph, we have the following remarkable assertion :

"From the statements of the physicians of Quito, and my own experience in its use, I am convinced that the 'condurango' is quite as reliable as a specific in cancer, scrofula, and other blood diseases, as cinchona and its alkaloid have proved to be in zymotic diseases."

The profession throughout the country will doubtless be anxious to verify the above when the supply of the remedy shall be ample, and when Dr. Bliss has vindicated himself before the public by the treatment of such cases as he may now have under his own observation. The profession will be patient.—[Med. Record.

NITRIC ACID IN BRIGHT'S DISEASE.—Dr. May Figueira, Physician to the Royal Hospital of St. Joseph, at Lisbon, has found great benefit from the use of pure nitric acid mixed with water (as lemonade) in Bright's disease. He gradually increases the dose to twenty-four and thirty drops four times a day. Milk and raw onions he found most useful in diminishing anasarca and albuminuria.

CHLOR-ALUM FOR UNBROKEN CHILBLAINS.—George P. Rugg, M. D., writes to the London Lancet that he has found an excellent remedy in the new disinfectant, chlor-alum, for unbroken chilblains. It should be applied, undiluted, night and morning, using a moderate amount of friction.

Solution of Ammonia in Delirium Tremens.—The efficacy of ammonia in drunkenness naturally suggests its employment in delirium tremens, which is only a prolonged attack of drunkenness, and it has recently been employed with good effects, in heroic doses, by Dr. Gouamier. One of his cases was a robust man, forty-five years of age, whose limbs were in a constant state of agitation, with sleeplessness and a sub-delirious state. Dr. Gouamier prescribed an ounce of strong infusion of valerian root, and thirty drops of solution of ammonia, edulcorated with simple syrup, to be taken every two hours ; after five doses, sleep supervened, and the agitation of the limbs was effectually cured.—[Gazetta Med. Lomb. et L'Imparziale, Jan. 1, 1871.—London Practitioner, Baltimore Reprint.

Subcutaneous Injection of Ergotin in Uterine Affections.—Dr. V. Swiderski recommends this method of treatment in cases of chronic metritis, displacements of the uterus, and metrorrhagia arising from various causes. He originally employed Bonjean's solution, which has the following composition : Ext. secal. corn. aquos. 2-5 ; sp. vin. rect. et glycerini, ana 7-5 ; but the pain occasioned was so great that he changed it for the following formula : Extract secal. cornut aquos. 1-0 ; sp. vin. rectif. 1-5 ; aq. destillat. 4-5, glycerini 3-0. A quantity of fluid, containing about 1-5 grains of ergot, was injected into the uterus on each occasion, and the injections were repeated every second day for a week or more. The effects obtained, especially in cases of metrorrhagia, appear to have been very satisfactory.

A mixture of pulv. steatite (soapstone), two parts, and hyd. chlor. mitis, one part, is the most elegant and effective dry application to the chafed skin of infants. Dry hyd. ch. mitis, applied once or twice a day to tumid and tender hemorrhoids situated about the anus, rarely fails to cure them in a few days.

To Prevent Pitting in Small Pox.—In a case recently treated, in which the eruption so completely covered the face that it was almost impossible to place the point of the finger on it, without touching it in one or more places, Dr. J. C. Whitehill, of St. Louis, Mo., (Med. Archives,) succeeded in absorbing the "pocks" completely, by anointing the face freely with a solution of carbolic acid, ℈j. and soda bisulph., ʒij., in an ounce of pure, fresh glycerine, and causing each vesicle, as soon as formed, to be punctured with a finely pointed hard wood, and some of the solution introduced. At the same time light was excluded, as far as possible, from the room, and a liniment of croton oil used over the chest as a revulsive. Not a "pit" was formed on the face.

Belladonna in the Treatment of Spermatorrhœa.—Mr. R. M. Jones recommends the employment of belladonna in so-called cases of spermatorrhœa consequent upon loss of tone and irritable state of the generative organs, as having accomplished very beneficial results in his hands, even in extreme cases. He generally prescribes it alone, in gradually increasing doses, until the desired effect is produced. He gives it, occasionally, in combination with quinine, or the tincture of the muriate of iron, but not with better results, as he has reason to believe that in some instances the iron has a tendency to neutralize the efficacy of the belladonna. He thinks the belladonna seems to possess a decided superiority over the iron in soothing the irritable state of the generative organs that is generally present in these cases. It also seems to possess some slight aphrodisiac qualities.

Treatment of Chilblains.—Mr. Fergus recommends sulphurous acid in this affection. It should be applied with a camel's hair brush. or by means of a spray producer. One application of this usually effects a cure. The acid should be used pure. A good wash for hands or feet affected with chilblains is sulphurous acid, 3 parts; glycerine, 1 part; and water, 1 part. The acid will be particularly useful in the irritating tormenting stage of chilblains.

Compound Solution of Iodine in Chronic Diarrhœa.—Dr. E. L. Shurly, Manistee, Mich. (Buffalo Med. and Surg. Journal,) writes that of all diseases abounding in that region he has found none more intractable or formidable than chronic diarrhœa, which is always of malarial origin. In the treatment of these cases all of the astringents and tonics of reputed efficiency, in connection with quinia, have been tried in vain. Driven by these unsatisfactory results to some new mode of action, he prescribed in several cases with effect, five drops of Lugol's solution in one-half tumbler of water, four times daily. The diarrhœa was entirely controlled by its use, and hepatic and splenic enlargements relieved.

Belladonna in Typhiod Fever.—Dr. Lewis S. Pilcher, Asst. Surgeon U. S. N.,) *Mich. Univ. Med. Journal,*) having been attracted by the positive and warm terms in which the effects of belladonna, given in typhoid fever, are stated by Dr. B. Kelly, of Dublin, finds that under its influence, within from 24–48 hours after the first administration of the drug, delirium, coma, and subsultus quickly vanish, and are succeeded by calmness and clearness of the intellect, by natural sleep, and complete control of all the voluntary muscles. Diarrhœa is checked, and healthy, consistant evacuations are established.—*Med. Record*, June 15, 1871.

Carbolic Acid as a Local Anæsthetic.—Dr. C. DuHadway of Jerseyville, Ill., (Med. Archives,) has beon using carbolic acid for the last two years as an anæsthetic in the removal of ingrowing toe-nails, also in abscesses, etc. He regards it as one of the best anæsthetics for these purposes, and uses the crystal in a state of dequilescence, full strength.

Hydrate of Chloral in Intermittent Fever.—Dr. F. A. Duzan, of Zionsville, Indiana, claims (*Indiana Journal of Med.* May, 1871) to have employed the hydrate of chloral successfully in the treatment of intermittent fever where quinia, arsenic, and other antiperiodics had failed. He gave fifteen grains five hours before the expected cold stage, and repeated the dose every hour until five doses had been taken. In one case narrated he repeated the doses on the day of the expected return, and after that there was no reccurrence of the disease.

Therapeutic Actions and Uses of Turpentine.—In the course of a paper on this subject (*Edinburgh Medical Journal*, July, 1871), Dr. Warburton Begbie takes occasion to recommend the use of turpentine in the severe headache which is apt to occur in nervous and hysterical women. " There is, moveover," he says, " another class of sufferers from headache, and this is composed of both sexes, who may be relieved by turpentine. I refer to the frontal headache, which is most apt to occur after prolonged mental effort, but may likewise be induced by unduly-sustained physical exertion,—what may be styled the headache of a fatigued brain. A cup of very strong tea often relieves this form of headache; but this remedy, with not a few, is perilous, for, bringing relief to pain, it may produced general restlessness and—worst of all—banish sleep. Turpentine, in doses of twenty or thirty minims, given at intervals of an hour or two, will not only remove the headache, but produce, in a wonderful manner, that soothing influence to which reference has already been made."

Soda Mint.—The very popular " Soda Mint," so much employed as an antacid and carminative for *over-fed* infants and dyspeptics, was originally a favorite prescription of Dr. Geo. Norris, of this city. His formula was the following:

| | | |
|---|---|---|
| ℞. | Sodæ Bicarb., . . . . . . | ℥ ss. |
| | Spt. Ammon. Aromat, . . . . . | ʒ j. |
| | Aquæ Menthæ Piperitæ, . . . | Oj. |
| M. | | |

Dose, from a desertspoonful to a tablespoonful for adults; from half to one teaspoonful for infants.—*American Joural Pharmacy.*

Carbolic Cerate.—The following will be an excellent formula for this preparation:

| | | |
|---|---|---|
| ℞. | Adipsis, . . . . . . . | ℥ x. |
| | Ceræ Albæ. . . . . . . . | ℥ v. |
| | Terebinth. Can., . . . . . . | ℥ j. |
| | Acid. Carbol., . . . . . . . | ℥ j. |

Melt the lard and wax together, add the balsam fir, and when it begins to cool stir in the carbolic acid.

The addition of balsam fir to this preparation corrects the disagreeable odor of the acid, and renders it slightly adhesive, which is quite desirable when the compound is used as a dressing for burns, old sores, &c.—*American Journal Pharmacy.*

Solution of Santonini:

| | | |
|---|---|---|
| ℞. | Santonini, in pulvere, . . . . | gr. xij. |
| | Sodæ bicarbonatis, . . . . . | gr. xx. |
| | Aquæ distillatæ, . . . . . . | ℥ iij. |

Put the soda and water into a flask, keep the fluid near the boiling-point, adding, as it disappears, about two grains of the Santonine at a time, until the whole is dissolved. Solution is affected in about half an hour, during which time the water is reduced by boiling, to ℥ ij. If need be, reduce by boiling, to this bulk, when ℥ j. will contain a full dose—six grains of Santonine. If an alkaline reaction be objectionable, neutralize with acetic acid.

In cases where powders are objected to, a mixture may be made by adding a little syrup and flavoring water to the Santonine solution.—*The Pharmacist*, April, 1871, from *London Practitioner.*

The After-taste of Quinine.—In practice there is often experienced a great difficulty in getting patients to take quinine, because of its after-taste, which, to some, is simply unbearable, and when antipathy thus exists, combined with a difficulty in swallowing pills, the therapeutic value of an important drug is lost. We find, and the fact may not be generally known, that the mastication of some acid fruit, as an apple or a pear, will permanently remove the disagreeable after-taste of quinine. The first mouthful of food should be well masticated and rolled through the mouth, so as to cleanse the teeth, etc., and then ejected. The second morsel may be swallowed, when it will be discovered that all taste of the quinine will be removed.—*Boston Med. and Surg. Journal, June 8th*, 1871, *from Med. Press and Circular.*

Artificial Food for Infants, can be made, which contains all the blood making and caloric generating properties of human milk. Wheat flour, however, has an acid reaction, and needs the addition of an alkali. By its use, also, it adds another task to the digestive organs of the infant, viz: that of converting the starch of the wheat into sugar. We may accomplish this process before adding the flour to the child's food, by mixing malt meal with the flour. When milk and wheat flour are boiled together and malt meal is added to the gruel, while hot, the mixture assumes a sweet taste. This reaction having taken place and an alkali having been added to neutralize the acid of the flour, we have essentially Liebig's food. Liebig's formula is:

℞. Wheat Flour.
Malt meal, . . . . . *aa* ℥ ss.
Bicarbonate Potassai, . . . grs. vii ss.

Mix with the aid of water ℥j, afterwards adding milk ℥v. Heat upon a slow fire and stirring until it gradually becomes thick, remove it from the fire for a few minutes, stir, and again place upon the fire, remove it as before, again place on the fire and leave it till it boils. Remove the bran by straining. German authors highly praise this preparation as a substitute for the mother's milk.—*Dr. E. V. Stodard, Buffalo Med. Jonrnal, July* 1871.

Chloral in Cancer.—Dr. W. Cooke commends most highly the use of chloral in cancer. Where there is persistent suffering, ten grains three times a day—otherwise, twenty grains at night—are the doses he has used in it.—*Medical Times and Gazette.*

Ice per Rectum in Chloroform Narcosis.—Dr. Baillie says (*L'Opinion Medicale*) that nothing is better in the narcosis of chloroform than the introduction of a morsel of ice into the rectum. A slight pressure on the sphincter relaxes it, the ice slips in, and immediately a deep inspiration is produced. This is the prelude to natural respiration and a restoration of the cardiac functions. He also recommends the remedy being tried on children born apparently dead.—*The Doctor.*

Injections of Calomel in Syphilis.—The *Revista Chinica di Bologna* relates eight cases of syphilis affecting the eyes, in which Dr. Soresina injected calomel suspended in glycerine. Six out of the eight cases were cured, and the other two somewhat improved. The successful cases included iritis, keratitis, retinitis, and paralysis of the third pair. The case in which the least effect was produced, was thought to be due to atrophy of the optic nerve.—*Ibid.*

Dr. Pancoast's Treatment of Inverted Toe-Nail.—Dr. Pancoast never removes the nail, nor any portion of it; but, as the trouble arises from the edge of the nail dipping down into the flesh at the side of the toe, he cuts away the soft parts, and leaves the nail in a position where it can do no harm; then, raising up its free edge, and separating it thoroughly from the parts below it, with a thin handle of a scalpel, he slips beneath it a strip or two of adhesive plaster, and carries the ends beneath the ball of the toe and round upon the metatarsus, so as to force the soft parts down and the nail up. When the parts heal, the side of the nail will be free from any covering. One great advantage of this operation is that the patient is almost immediately enabled to attend to his business. He keeps the parts covered, for several days, with a strong aqueous solution of sub-acetate of lead and laudanum.—*Medical Archives.*

DRESSING OF WOUNDS WITH TINFOIL.—Dr. Florchutz states, in the *Med. C. Zeit.*, Jan. 4, that he had lately under his care numerons wounded German soldiers, who reached the hospital after six days' journey. At first he used the common dressings, but soon gave the preference to tinfoil. The latter is placed, in an imbricated manner, on the wound, and over the foil some charpie where the suppuration is abundant, and a simple compress where it is not. The dressings were changed twice in the twenty-four hours at first, but only once when the patients improved. The author prefers the tinfoil to lead-leaf, which had been recommended, and has found that the most severe gunshot wounds did well with the tinfoil, which latter is very cheap, and easily procurable.—*Lancet.—Ibid.*

MANAGEMENT OF SCABIES.—Dr. M'Call Anderson (*Richardson's Hand-Book of Microscopy*) grealy prefers, in the treatment of scabies, in private practice, the following formula:

℞. Styracis liquid, . . . . . . . ℥ j.

Adipsis, . . . . . . . . ℥ ij.

Melt and strain.

This is a clean-looking ointment, has a pleasant aroma, kills the acari, and does not irritate the skin in the least, but, on the contrary, rather soothes it.

CASTOR OIL MADE PALATABLE.—A correspondent of the *Boston Medical and Surgical Journal*, suggests that this article of the Pharmacopœia may easily be made palatable by the employment of glycerine as an excipient; in fact, the dose will be found as "sweet as honey," and devoid of any unpleasant taste:

℞. Glycerine (puriss).

Ol. ricini, . . . . . . . *aa* ℥ ij

Ol. cinnamoni, . . . . . . *m* iv.—M.

The ol. cinnam. should be rubbed up with the glycerine, and the ol. ricini then added, and the whole well mixed, by being shaken, when used. In larger doses, lessen the proportion of the glycerine.

Rigid os Uteri.—A quarter of grain of morphia hypodermically injected in cases of tedious labor from rigid os uteri, has the effect of producing rapid dilatation, in addition to its soothing effects upon the worn out and suffering system generally.—*Virginia Clinical Record.*

Tetanus Neonatorum Treated with Chloral Hydrate.—Dr. Widerhofer, of Vienna, showed lately to his class a child three months old, which was attacked by tetanus neonatorum at the end of the first week after birth, and was treated with chloral hydrate in doses of one and two grains at the time of each onset of convulsions. It was in danger for a fortnight. During the intermission of the spasms it was fed from the breast by its mother. It is now a fine, healthy-looking child. This is the sixth case (out of ten or twelve) that Dr. Widerhofer has had of recovery under treatment by chloral. Under all other methods all his previous cases died. Considering that Vogel, and other great German authorities on children's diseases, had quite recently never seen a case of this affection recover, such a success must be taken to indicate a real advance in therapeutics. Dr. Widerhofer gives from two to four-grain doses of chloral by the rectum, if the infant cannot take it by the mouth.—*London Lancet.*

Glycerin Inhalations in Croup.—Dr. Stehberger, of Mannheim (*American Practitioner*), recommends in the treatment of croup the inhalation of glycerin by atomization. It increases the secretions of the mucous membrane, and softens the cough. The inhalation is to be continued for fifteen minutes, and repeated in half an hour, or whenever necessary. It is of no service in the advanced stage.—*Pacific Med. & Sur. Journ.*

Chloral Hydrate in Enuresis.—A writer in the *British Medical Journal* recommends this article in the highest terms for the cure of incontinence of urine. A girl who had suffered nightly for weeks took fifteen grains every night before retiring, and not a single return of incontinence took place.

Hydrate Chloral in Whooping-Cough.—Dr. A. M. Fountelney, (Richmond and Lou'sville Medical Journal,) says: We have employed the chloral hydrate in whooping-cough, combined with bromide of potassium. The intervals between the paroxysms of coughing have lengthened, and the severity of cough abated. To children between three and four years old we have given the following:

℞. Chloral Hydratis, } aa., ℈ j.

Potassii Bromid, }

Syr. Pruni Virgin., ℥ ss.=2½ oz. M.

Sig.—Dose, teaspoolful every three or four hours.

We have combined the chloral hydrate with hydrocyanic acid and bromide of potassium with apparently good effects in whooping-cough. We believe that chloral hydrate may be made to play an important role in infantile therapeutics.

Cod-Liver Oil Made Palatable.—Dr. Hager states that the addition of ten drops of chloroform to 100 grammes of cod-liver oil renders that oil perfectly agreeable to the palate without in the least impairing its good quality as a medicine, or interfering with its effect on the system.—*Neues Jahrbuch fur Pharm.*

Muriate of Quinia in solution of 25 ctgrm. in grm. distilled water (gr. iv to ℥ i) is recommended by Italian physicians as an excellent collyrium in chronic catarrh of the conjunctiva, &c.—*Pharm. Zeitung.*

Chloral hydrate and Cod-liver Oil.—10 grm. crystallized pure chloral hydrate dissolved by digestion in a sand bath, in 190 grm. cod-liver oil, renders the latter more palatable; used by consumptives, the preparation diminishes the night-sweats, produces sound sleep and improves the appetite. The dose is six tablespoonfuls daily.—*Pharm. Zeit.* from *Gaz. Phaam. Ital.*

Treatment of Enlarged Tonsils.—Dr. Rumbold, St. Louis, Mo., (*Med. Archives*,) has treated, successfully, a number of cases of enlarged tonsils, by injecting the glands by means of a hypodermic syringe, with a solution of iodine—iodine gr. ij., potass. iod. ℈ ij., aquæ ℥ j. Generally a slight inflammation followed the injection, but soon sudsided. From 12 to 17 injections—ordinarily two a week—were sufficient to reduce the gland to its normal condition. The advantage claimed for this mode of treatment was, saving the substance and function of the gland.

Iodide of Ammonium Preferred to Iodide of Potassium.—Dr. J. W. Curran (Medical Press and Circular) is confirmed in the belief that iodide of ammonium is more potent in therapeutics than iodide of potassium, He gives it the preference in the treatment of glandular affections, and extols it highly in cutaneous erysipelas. His method of applying it in erysipelas is in the form of ointment spread on lint, as well as internally. The ointment is composed of thirty grains of the iodide to an ounce of simple cerate. He says it rapidly promotes absorption of the effusion underneath the skin, and has been uniformly successful in sixteen cases. He also gives, internally, four grains three times a day, with infusion of cinchona. "I am proud to say," he continues, "that the rash has never spread beyond the anointed lint."—*The Med. Record.*

Hypodermic Medication.—Dr. T. Curtis Smith, Middleford, Ohio, gives the following directions for using the Hypodermic Syringe:

Having a proper solution, the syringe is charged with the proper dose, the skin is pinched up between the thumb and finger, the point of the needle is placed against the integument at the point where the needle is to enter. It is then thrust through the skin by a quick motion, after which it is carried one inch under the skin, but quite parallel to the surface. We are now directed by authors on this subject, to discharge the fluid drop by drop, until the requisite amount is injected. But as the only great danger from hypodermic injections is that of either using too large a dose or of entering a vein, I think these directions can be much improved upon. I present my own plan of using, and let the reader judge for himself whether or not it is an improvement on the plan usually adopted.

The needle is carried one inch under the integument into the cellular tissue, but instead of injecting the fluid at that point, the needle should be withdrawn a quarter or half-inch; then, holding the barrel of the syringe parallel with the surface, raise the skin three to five lines above the surrounding surface. If the integument is very loose, it can be raised higher than that without endangering the integrity of the needle. After it is thus managed, the fluid can be injected very slowly. If a vein has bean entered, the partial withdrawal will, most likely, dislodge it from its channel; but if it does not, the little force used to raise the integument will rupture the very thin walls of so small a vein as is found so near the integument; and, whether punctured or ruptured, it will be pouring out its own blood while the solution is being absorbed, thus preventing the medicine from being carried directly to the heart. It is my opinion that by carefully following these directions, the injections of a vein will nearly always be avoid. I doubt whether one could be injected by following this plan.

The syringe should contain no air. If air is drawn into the syringe with the solution, it can readily be inverted and pressed out by pushing in the piston. A minim or two more should be carried into the barrel, as one minim will be left in the barrel and needle. Besides, if a little air be left in the barrel, an overplus of one or two minims gives opportunity to avoid pressing the piston entirely home, thus avoiding throwing the air into the cellular tissue.—*Med. Surg. Reporter.*

A New Test for Hysteria.—A French work just issued by Dr. Chalron, Chief Medical Officer to the Vesinet Asylum, entitled "Clinical Studies on Hysteria," announces the discovery by him, of a new pathognomonic sign of hysteria, which, should it be confirmed by experience, will prove to be a valuable contribution to medicine. Since Dr. Chairon has become connected with the Institution, he had passed under view 26,000 female patients, amongst whom were a great many cases of hysteria. He says that he has ascertained that in every one of them the commencement of the affection has been marked by a special sign—insensibility of the epiglottis.

The determination of this symptom, which is constantly present, is very simple. It is sufficient to introduce gently the finger into the mouth, so as not to frighten the patient, and place it on the base of the tongue. It will be found that the epiglottis may be touched, displaced and scratched with the nail without producing the least regurgitation. When this symptom exists there will be found invariably a congestion of one or both ovaries, usually of the left.

Singular as this proposition is, the author proceeds to prove its exactitude, and has, with that object, quoted a great number of cases collected at Veinet.

TREATMENT OF INTERMITTENT FEVER BY CARBOLIC ACID.—(Translated by Dr. H. Tuck, from Wein. Med. Presse, March 19, 1871).—Dr. Treulich reports eight cases of intermittent fever promptly cured by carbolic acid. His formula is:

| | |
|---|---|
| ℞.—Acidi carbolic, (cryst.) . . . | gr. iij, |
| Inf. gent, . . . . . . . | ℥ v. |
| Syr. simpl., . . . . . . | ℥ i. |
| M. Dose, ʒ i. ter die. | |

His article closes thus:

1. Carbolic Acid is an admirable remedy for intermittent fever, even for obstinate cases which have resisted quinine.

2. Its action is speedy and certain, and it requires such a small amount that it cannot possibly have any injurious effect on the system.

3. The average amount required was four and one-eighth grains.

4. It costs only one thirty-fifth of what quinine does, and so is to be preferred for the poor.

5. This successful use of carbolic acid proves that the action of quinine in intermittent fever is antiparasitic.

6. It also favors the opinion that intermittent fever is the result of a blood poison.

IN capillary bronchitis of infants, Prof Abelin, of Stockholm, extols steam. Patients should remain in the vapor for days, and even weeks. This treatment is also useful in pneumonia.—*Jour. f. Kilderheilkunde*

SALICINE IN TYPHOID FEVER is highly recommended by some German practitiner. It is said to do good as an antiseptic.

TO MAKE BENZOATED OINTMENT :

| | | |
|---|---|---|
| R.—Benzoin pulv. (select, | . , . | oz. ij. |
| Ether Sulphuric, | . . . | oz. iv. |
| Ol. Ricini, | . . . . . | oz. j. M |

Introduce the benzoin into an eight ounce bottle, add the ether, macarate for twenty-four hours with frequent agitation, pass through a filter, to the filtrate add ol. ricini, and shake until dissolved, then transfer to a shallow vessel in oxder to allow the ether to evaperate spontaneously ; lastly. when the ether has entirely disappeared, place in a wide-mouthed bottle ready for use. —(C. F. Bolton, in *Amer. Jour. of Pharmacy.*

THE following preparation of cod liver oll will be found to be quite generally acceptable :

| | | |
|---|---|---|
| R.—Cod Liver Oil, | . . . | 4 ounces. |
| Citrate Iron and Quinine, | . . | 1 drachm. |
| Extract Bitter Almonds, | . . | 1 drachm. |
| Glycerine, | . . . . . | 2 ounces. |

Mix.

S.—Dose a dessert spoonful.

PSORIASIS OF TWENTY YEARS' STANDING CURED BY IODIDE OF POTASSIUM.—Charing Cross Hospital ; treatment continued for six weeks ; later doses given during three weeks of treatment.—*Med. Times & Gazette, May* 20.

VERATRUM VERIDE IN PUERPERAL CONVULSIONS.—Herbert Fearn, M. D., records ten cases of his own, and three in the practice of others, to show that veratum viride in large doses "is a complete substitute for copious blood-letting. * * The quantity should be regulated by the condidition of the case. * * If the case is very mild, fifteen drops may be sufficient, if repeated often enough. * * If the case be a very violent one, * * I would give one, two, or more drachms of Norwood's tincture within five or ten minutes, * * there being no danger from the medicine so long as the convulsions continue. When the pulse becomes soft, yielding, and reduced to about forty-eight, * * or when severe vomiting occurs it will generally be advisable to stop the medicine, at least for a short time, and watch the effects. If the pulse manifests a tendency to go below forty-eight, it will be best, in the mild cases, to give a quarter of a grain of morphine, and repeat often enough to stop the vomiting. * * I believe the veratrum has a direct sedative effect over the spinal cord, but think, on the other hand, its primary influences is on the ganglionic or sympathetic nervous centres."—*Amer. Journ. Obstetrics, May.*

TREATMENT OF VARICOSE VIENS.—Mr. Henry Smith, of King's College Hospital, London, (Am. Practitioner,) adopts the old plan of obliterating varicose veins by acupressure with many pins. As many as ten perhaps are passed well under the vein,, at distance of two or three inches apart, with a hare-lip suture placed above. The operation is quite painless, and such cases are treated as out-patients.

ACUTE INFLAMATION OF THE PROSTRATE GLAND, DR J. H. MCTAGGART, OF PHILADELPHIA, SAYS:—Acute inflammation of the prostrate gland is often the result of gonorrhœa or blows, and when it takes place runs its course with extreme rapidity.

In order to obviate that difficulty, and control the condition of local depression, we have found, in a very extensive city practice, the following to be excellent:

℞. Tinct. green root of gelseminum, . ℥ ss
Aqua, dest., . . . . . . . ℥ iv.
Acetate postassæ, . . . . . . ʒ vi. *Mix.*

A teaspoonful every hour, until the physological effects of the gelseminum are visible, then one every four hours. Hold this position, once gained, for several days, when nature is aided to overcome the condition; then follow up with:

℞. Fluid ext. hydrangea, . . . . . ℥ iv.
Iodide potass., . . . . . . ʒ ii. *Mix.*

In teaspoonful doses.

I found the above a good way of managing this complication.—*Pennsylvania Eclectic Medical Journal.*

DIARRHŒAS of the simple kind, says Dr. Adolphus, and even those of an inflammatory character, have been best treated by me with carbolic acid, 20 grains to the ounce; teaspoonful every 12 hours in water. I have always successfully cured diarrhœa in this way. One precaution is needed; the patient's diet must be regulated; albuminous food must not be taken near the time the medicine is—an hour should elapse.

Another splendid cure for diarrhœa, is:

℞. White rosin, . . . . . . 3 lbs.
Turpentine, . . . . . . . 1 qt.
Balsam fir, . . . . . . ʒ iv. *Mix.*

Melt the rosin and add the others.

*Dose*—From 10 to 30 drops, on sugar, as often as needed. Catarrh of the stomach has yielded to this in my hands more often than to any other remedy; so has catarrh of the bowels and bladder.—*Ibid.*

Painful Menstruation, with or without menorrhagia, is met successfully by the cannabis indicus. The stuff in the market sold as fluid ext. of the article is like a great many others of the kind —a swindle. I have found few reliable, as a general thing, and always use the English solid extract when obtainable. This medicine has a specific influence on the uterus in a marked degree, curing menorrhagia, painful mensuration, uterine engorgements and neuralgia of the organ. I use it in full doses, because I desire its full anodyne and sedative effect on the organ.

Ergot and veratum viride make a valuable combination in many forms of neuralgia, and other conditions induced by feebleness of the vasa motor system. The difficulty of getting a good article of ergot embarrasses the phyaician. When the remedy is six months old, and exposed to the air in jars, with only a loose cover on them, or in paper packes, the certainty of its deterioration is fixed. It should be made up at once into tincture. I have kept ergot a year in a well-sealed bottle and found it good when used, but somewhat feebler than the recent berry. Exposure to the light damages the tincture also, as I have found a heavy sediment when so exposed a few weeks, and the tincture of less therapeutic value. Heat damages it to a great extent.

Ergot has the best influence in heat diseases of any other remedy I have found. Ten drops of the saturated tinct., every four hours, is a dose until the urgent symptoms pass off. It is far superior to digitalis. In pneumonia I think it of far exceeding value. Even in the low forms of continued fever, where there is coma or delirium, from a feebleness of the vasa motor and the capillary circulation, we have in it a grand remedy of great value. I have seen ergot do surprisingly good in epilepsy and paralysis of some kinds. It deserves a very extended experimented trial in fevers and inflammations, as well as in nervous disorders. I have seen it work like magic in both fevers and neuralgic affections. I have given it in drachm doses in severe and painful neuralgias, repeated every hour until the pain is controlled. It aids the action of quinine remarkably. I always, when I can, use the saturated tincture made from the recent berry.—*Ibid.*

